# Modulatory Effects of Water Extract from *Eleutherine americana* on Intestinal Health: Insights from Growth, Morphology, Microbiota and Metabolomics in *Plectropomus leopardus*

**DOI:** 10.3390/ani16213359

**Published:** 2026-10-24

**Authors:** Hongbiao Dong, Xiangbing Zeng, Yongchao Hu, Xiaoting Zheng, Jiasong Zhang

**Affiliations:** 1 Key Laboratory of South China Sea Fishery Resources Exploitation & Utilization, Ministry of Agriculture, South China Sea Fisheries Research Institute, Chinese Academy of Fishery Sciences, Guangzhou 510300, China; donghongbiao@163.com (H.D.); xiangbing1998@163.com (X.Z.); xtzheng1990@163.com (X.Z.); 2 Sanya Tropical Fisheries Research Institute, Sanya 572000, China; 3 Key Laboratory of Efficient Utilization and Processing of Marine Fishery Resources of Hainan Province, Lingshui 572426, China; 4 College of Marine Biology and Fisheries, Hainan University, Haikou 570100, China; yongchaohu2001@163.com

**Keywords:** *Eleutherine americana*, *Plectropomus leopardus*, intestinal health, survival, histomorphology, metabolomics, metagenomics

## Abstract

This study evaluated dietary *Eleutherine americana* water extract (EAW, 0 [NC], 1 [LD], 5 [MD], and 10 [HD] mL/kg) in 480 juvenile leopard coral grouper (*Plectropomus leopardus*; initial body weight, 11.72 ± 0.34 g) over 8 weeks, with four replicate tanks of 30 fish per treatment. EAW did not significantly improve growth performance, but survival was higher in MD and HD than in NC (*p* < 0.05). Histological observations indicated more intact mucosal folds, greater crypt depth, and higher goblet-cell counts. Metabolomic analysis showed lower carnitine levels and higher phospholipid and prostaglandin metabolites, with annotated differences in bile acid biosynthesis, amino acid metabolism, and carbon metabolism pathways. Intestinal microbiota profiles showed lower relative abundances of Pseudomonadota and Basidiomycota in EAW groups, lower Actinomycetota in LD and MD, and higher Bacteroidota and Bacillota in MD. At the genus level, *Bradyrhizobium*, *Microbacterium,* and *Sphingobium* abundances were lower, and *Afipia* abundance was higher, in EAW groups. Functional profiles varied in carbohydrate, amino acid, and lipid metabolism categories. Microbiota–metabolite correlation networks identified Pseudomonadota, Actinomycetota, hexosylceramides, and carnitines as highly connected features. Network pharmacology identified *pdgfra*, *pdgfrb*, and *sirt1* as candidate targets of the major EAW components eleutherol, eleutherin, and isoeleutherin. The survival and intestinal findings support further evaluation of EAW as a feed additive for intestinal health in leopard coral grouper.

## Simple Summary

Maintaining intestinal health is important for the survival of farmed fish. Plant extracts may help support gut function when added to feed. This study examined a water extract of the medicinal plant *Eleutherine americana* in juvenile leopard coral grouper, an economically important marine fish. In an eight-week feeding trial, 480 fish received diets containing different amounts of the extract. Survival was higher at 5 and 10 mL/kg of feed than with the unsupplemented diet, with the highest observed survival at 5 mL/kg. Among the fish surviving to the end of the trial, supplemented groups showed a more intact intestinal lining, together with changes in gut microorganisms and small molecules involved in nutrient processing. These findings suggest that intestinal protection may contribute to the survival benefit associated with the extract. The study supports *E. americana* as a feed additive to promote intestinal health and support the farming of leopard coral grouper.

## 1. Introduction

The leopard coral grouper (*Plectropomus leopardus*) belongs to the order Perciformes, family Serranidae, subfamily Epinephelinae, and genus *Plectropomus*. It is distributed in tropical and subtropical waters of the Indo-Pacific and is an economically important aquaculture species [1]. Its growth, physiological stress responses, and coloration have been investigated in relation to environmental conditions, handling, and dietary supplementation [2,3,4], and genomic resources have been developed to support selective breeding [5]. Bacterial infection and enteritis remain important health concerns during cultivation [6,7], creating a need for feeding strategies that support intestinal health.

Chinese herbal feed additives have been studied as alternatives or complements to conventional disease-management strategies in aquaculture [8]. *Eleutherine americana* (Aubl.) Merr. ex K.Heyne (Iridaceae) is a synonym of the accepted name *Eleutherine bulbosa* (Mill.) Urb.; *Eleutherine plicata* (Sw.) Herb. is also listed as a synonym [9]. Isoeleutherin from *E. americana* suppresses inducible nitric oxide synthase and NF-κB signaling in stimulated macrophages [10], while a crude extract showed antioxidant and antibacterial activity in a cooked-pork model [11]. *E. americana* preparations also affected bacterial growth in cultures of human intestinal microbiota and increased short-chain fatty acid production in infant-derived microbial cultures [12,13]. These results support investigating dietary *Eleutherine* preparations for intestinal health.

Dietary *Eleutherine* preparations have been investigated in aquatic animals. In striped catfish (*Pangasianodon hypophthalmus*), Nugroho et al. evaluated *Eleutherine bulbosa* extract at 15–60 g/kg feed and reported significantly higher final body weight and average weekly weight gain at 30–60 g/kg, together with increased amylase activity at 45 g/kg [14]. In white shrimp (*Litopenaeus vannamei*), Munaeni et al. compared *E. bulbosa* powder at 6.25–25 g/kg with crude extract at 1.25 g/kg. The 12.5 and 25 g/kg powder treatments increased protease, lipase, and amylase activities, intestinal perimeter ratio, and microvillus density. Intestinal bacterial diversity was highest in shrimp receiving 12.5 g/kg powder [15]. A separate study showed that 12.5 g/kg *E. bulbosa* powder increased total hemocyte counts, phenoloxidase activity, and respiratory burst activity, reduced bacterial loads following *Vibrio parahaemolyticus* challenge, and improved post-challenge survival [16]. In laying hens (*Gallus gallus domesticus*), dietary supplementation with 10 g/kg Dayak onion powder, reported as *Eleutherine palmifolia*, significantly decreased fecal pH and Enterobacteriaceae counts while increasing lactic acid bacterial counts [17].

Enteritis in leopard coral grouper has been associated with altered intestinal microbiota and inflammatory and metabolic responses [7]. Maintaining intestinal health is therefore an important goal in culture. However, the effects of *E. americana* on intestinal health in leopard coral grouper have not been investigated. This study examined the effects of dietary *E. americana* on intestinal metabolites and microbiota and used network pharmacology to investigate candidate mechanisms.

## 2. Materials and Methods

### 2.1. Preparation of Water Extract from E. americana (EAW)

Fresh stems of *E. americana* were washed with distilled water, dried in a forced-air oven at 50 °C for 2 days, and ground into a fine powder using a laboratory blender. Dried stem powder (500 g) was mixed with 1000 mL distilled water at an initial material-to-solvent ratio of 1:2 (*w*/*v*). The mixture was extracted at room temperature for 24 h with continuous magnetic stirring, then vacuum-filtered at room temperature through Whatman filter paper (diameter: 125 mm; Whatman International Ltd., Maidstone, UK). The final filtrate volume was 900 mL. Each milliliter represented approximately 0.556 g of starting dry stem material, corresponding to 0.556, 2.778, and 5.556 g starting material/kg feed at inclusion levels of 1, 5, and 10 mL/kg, respectively. The water extract of *E. americana* (EAW) was analyzed by liquid chromatography–quadrupole time-of-flight mass spectrometry (LC-QTOF-MS); the main constituents are listed in Table S2.

### 2.2. Fish Collection and Acclimation

Leopard coral grouper were obtained from Lingshui Delin Chengxin Aquaculture Co., Ltd. (Hainan, China) and acclimated for 14 days in indoor experimental tanks at the Tropical Fisheries Research Center, South China Sea Fisheries Research Institute, CAFS. During acclimation, water temperature was maintained at 28 ± 1 °C, pH at 7.8 ± 0.3, salinity at 25–28 ppt, and dissolved oxygen at ≥7.0 mg/L under a natural photoperiod. Water quality was monitored daily, with 0.2 mg/L ammonia nitrogen as the management threshold. Intermittent water exchange typically replaced approximately 70% of the water volume per exchange; the proportion was adjusted according to ammonia–nitrogen readings. Fish received a commercial diet (Haitong Feed Co., Ltd., Zhanjiang, China), whose nutritional composition is given in Table S1.

### 2.3. Fish Culture and Sampling

After the 14-day acclimation, fish with an initial mean body weight of 11.72 ± 0.34 g were randomly assigned to four dietary treatments: NC (control, 0 mL/kg EAW), LD (1 mL/kg EAW), MD (5 mL/kg EAW), and HD (10 mL/kg EAW). Each treatment had four replicate circular tanks (500 L; bottom area, 1 m^2^), with 30 fish per tank and 480 fish in total. The stocking density was 0.7 g/L. EAW was sprayed evenly onto the Haitong commercial diet at the assigned doses immediately before feeding; the control diet received an equal volume of distilled water. Fish were fed twice daily (07:30 and 17:30) to apparent satiation, with the daily ration adjusted between 2% and 5% of body weight according to observed consumption. Husbandry and environmental monitoring during the 8-week feeding trial followed the same procedures as during acclimation. After 8 weeks, survival rate (SR), weight gain rate (WGR), specific growth rate (SGR), and condition factor (CF) were calculated as follows:

SR = N_F_/N_I_ × 100%

SGR = [ln(W_F_) − ln(W_I_)]/T × 100%

WGR = (W_F_ − W_I_)/W_I_ × 100%

CF = W_F_/L^3^ × 100%

N_I_ and N_F_ are the initial and final numbers of fish, respectively; T is the feeding duration (56 days). W_I_ and W_F_ are the initial and final mean body weights of the juvenile leopard coral grouper, respectively. L is fish body length (cm).

After a 24 h fasting period at the end of the feeding trial, four surviving fish were randomly sampled from each replicate tank, giving 16 fish per treatment group. Histology, metabolomics, and metagenomics used the same four fish from each tank, and the sampling site was the hindgut. Intact hindgut segments from each fish were fixed and embedded separately for histology and were not pooled. For metabolomics, equal masses of hindgut wall tissue from the four fish in each tank were pooled into one composite sample. For metagenomics, hindgut contents from these fish were combined into one composite sample per tank. Thus, each omics analysis used four tank-associated composite samples per treatment. Samples for metabolomics and metagenomics were immediately flash-frozen in liquid nitrogen.

### 2.4. Histopathology Analysis

Intact hindgut segments from each fish were immersion-fixed separately in 10% neutral buffered formalin, rinsed with ice-cold saline, and dehydrated through a graded ethanol series (70% to 100%). Tissues were cleared in xylene and embedded separately in paraffin wax. Serial sections (5–6 μm) were cut with a microtome, mounted on glass slides, and stained with hematoxylin and eosin (H&E). Images captured under a light microscope were used to measure mucosal fold length and width, muscularis thickness, mucosal thickness, crypt depth, and goblet-cell count. At least five intact, straight mucosal folds were randomly selected from each section for these measurements. For each histological parameter, measurements from the sections of each fish were averaged to obtain a mean value for that fish. The four fish means within each tank were then averaged to obtain one tank mean for comparison among dietary treatments.

### 2.5. Metabolomics Analysis

Intestinal samples were cooled on ice, homogenized, and centrifuged at 15,000 × g for 20 min. The supernatant was collected, diluted with LC-MS-grade water to a final methanol concentration of 53%, transferred to new tubes, and centrifuged again. Metabolites were separated and detected using a Thermo Fisher Vanquish UHPLC system (Thermo Fisher Scientific, Germering, Germany) coupled to an Orbitrap Q Exactive™ HF high-resolution mass spectrometer (Thermo Fisher Scientific, Bremen, Germany). Raw data were processed in Compound Discoverer 3.1 (Thermo Fisher Scientific, San Jose, CA, USA) for peak alignment and peak picking. Metabolites were annotated against mzCloud, mzVault, and MassList, with further annotation using KEGG, HMDB, and LIPIDMaps.

Metabolomic statistical analyses were performed in R. MetaboAnalystR (version 4.2) was used for normalization to approximate a normal distribution, principal component analysis (PCA), and orthogonal projections to latent structure-discriminant analysis (OPLS-DA). Differentially abundant metabolites (DMs) were identified by a univariate *t*-test using *p* < 0.05 and an absolute log_2_(Fold Change) greater than 1. The randomForest package (version 4.7) ranked metabolites by feature importance. Heatmaps of Z-score-normalized data were generated in TBtools (version 2.056), and DMs were subjected to over-representation analysis (ORA) of KEGG pathways.

### 2.6. Metagenomic Analysis

Total DNA was isolated from hindgut contents using a modified extraction protocol involving cetyltrimethylammonium bromide (CTAB, Shanghai Aladdin Biochemical Technology Co., Ltd., Shanghai, China) and sodium dodecyl sulfate (SDS, Shanghai Aladdin Biochemical Technology Co., Ltd., Shanghai, China). DNA concentration and integrity were assessed using a NanoDrop One spectrophotometer (Thermo Fisher Scientific, Wilmington, DE, USA) and 1% agarose gel electrophoresis. DNA samples that passed quality checks were used to prepare shotgun metagenomic libraries with the NEBNext Ultra DNA Library Prep Kit (New England Biolabs, Ipswich, MA, USA) for Illumina, with unique index codes for multiplexing. Library quality and molarity were assessed using a Qubit 2.0 Fluorometer (Invitrogen, Carlsbad, CA, USA) and an Agilent Bioanalyzer 2100 system (Agilent Technologies, Santa Clara, CA, USA). Libraries that passed quality checks were sequenced on an Illumina NovaSeq platform (Illumina, San Diego, CA, USA) using a paired-end configuration of 2 × 250 bp. Adapter sequences and low-quality reads were removed using the sequencing facility’s quality-control pipeline. Host-derived reads were removed when a reference genome was available.

The quality-controlled reads were taxonomically classified using Kraken2 (version 2.17.1) [18]. Relative abundances at the genus and species levels were estimated using Bracken (version 3.1) with the corresponding Kraken2 database [19]. Functional profiling used HUMAnN 3 (version 3.9) with the UniRef90 database [20]. UniRef90 gene families were regrouped into KEGG Ortholog abundances and summarized into KEGG pathway categories. Taxonomic and functional abundance tables were normalized. Microbial co-occurrence networks were constructed from taxonomic abundance profiles using pairwise Spearman correlations and visualized with igraph (version 2.2.1) in R and Gephi (version 0.9.7).

### 2.7. Correlation Network Analysis of Intestinal Microbial Taxa and Host Metabolites

Correlations between taxonomic features and DMs were calculated with the MetaNet R package (version 0.3.2). Pairs with Spearman |r| > 0.6 and *p* < 0.05 were included in the microbiota–metabolite network.

### 2.8. The Regulatory Gene of EAW on Intestinal Health

We used network pharmacology and molecular docking to examine candidate mechanisms of EAW activity in the intestine. Canonical SMILES for five components, citric acid, benzoic acid, eleutherol, isoeleutherin, and eleutherin, were retrieved from PubChem (https://pubchem.ncbi.nlm.nih.gov/, accessed on 15 October 2024) and submitted to SwissTargetPrediction (https://www.swisstargetprediction.ch/index.php, accessed on 15 October 2024) and TargetNet (http://targetnet.scbdd.com/, accessed on 15 October 2024). Targets with probability scores greater than zero were retained. Enteritis-related targets were retrieved from GeneCards, OMIM, and TTD using the keyword “Enteritis”. The target lists were combined, and gene names were standardized to official symbols using UniProt. Targets shared by the EAW components and enteritis were used to construct a protein–protein interaction (PPI) network in STRING (https://cn.string-db.org/, accessed on 15 October 2024). Their functional associations were examined in the biological context of leopard coral grouper, with GO enrichment and KEGG pathway analyses. Three-dimensional structures of Pdgfra, Pdgfrb, and Sirt1 were obtained through AlphaFold Server (https://alphafoldserver.com/, accessed on 16 October 2024). Molecular docking used AutoDock (version 15.7.6) to predict binding modes and affinities between the EAW components and these proteins. Protein structures were prepared by adding polar hydrogen atoms and assigning charges. Each ligand–protein pair underwent 50 independent docking runs. The pose with the lowest binding free energy was selected using a semi-empirical free-energy force field that accounts for the estimated change in conformational entropy on binding.

### 2.9. Statistical Analysis

Data are presented as mean ± standard deviation. Statistical analyses were performed in SPSS 22.0. Normality and homogeneity of variances were assessed using the Shapiro–Wilk and Levene tests, respectively. One-way analysis of variance was followed by Duncan’s multiple comparisons, with significance set at *p* < 0.05. Tanks were the independent experimental units, with four replicate tanks per treatment (*n* = 4). For histomorphometry, section measurements were averaged within each fish, and the fish means were then averaged within each tank. The four tank means per treatment were used for comparisons among dietary treatments.

## 3. Results

### 3.1. Main Components of EAW

Chromatographic and mass spectrometric analyses annotated phenolic and organic acid derivatives among the constituents of EAW. The annotated compounds included citric acid (C_6_H_8_O_7_), benzoic acid (C_7_H_6_O_2_), and multiple eleutherol isomers (C_14_H_12_O_4_), along with structurally related derivatives such as isoeleutherin and eleutherin (C_16_H_16_O_4_) (Table S2).

### 3.2. Growth Performance of Leopard Coral Grouper by Dietary EAW

Final body weight, weight gain rate, and specific growth rate were slightly higher in LD and MD than in NC, but the differences were not significant (*p* > 0.05). Survival was significantly higher in MD and HD than in NC (*p* < 0.05). Condition factor did not differ significantly among groups (*p* > 0.05; Table 1).

### 3.3. Histopathology Alteration of Leopard Coral Grouper by Dietary EAW

**Figure 1:**
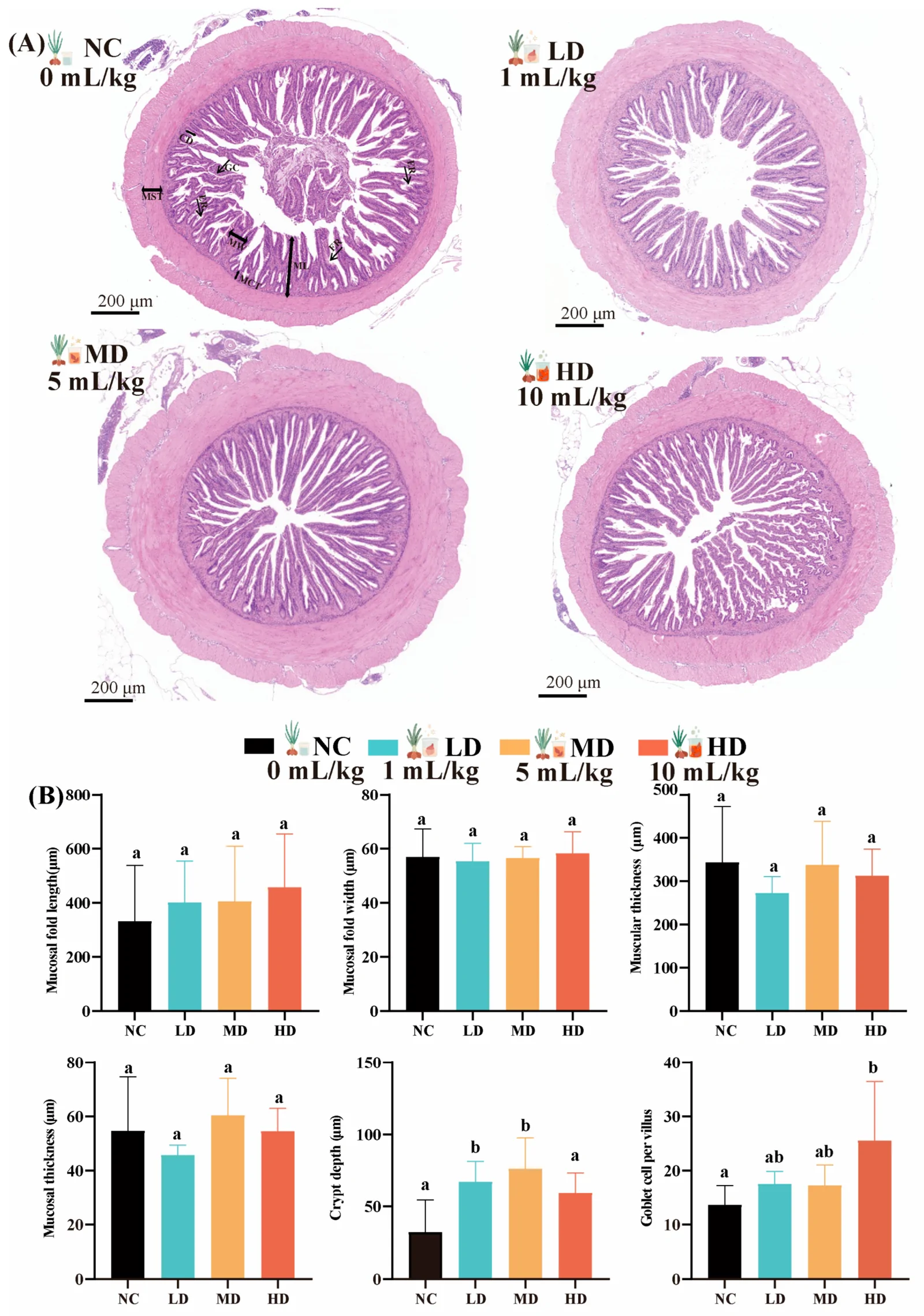
Intestinal histopathology of leopard coral grouper fed dietary EAW. (**A**) Representative intestinal sections from NC, LD, MD, and HD. ML, mucosal fold length; MW, mucosal fold width; MST, muscular thickness; MCT, mucosal thickness; CD, crypt depth; GC, goblet cell; ER, epithelial rupture. (**B**) Mucosal fold length and width, muscular thickness, mucosal thickness, crypt depth, and goblet cells per mucosal fold. Different superscript letters indicate significant differences (*p* < 0.05). Scale bars in panel A: 200 μm. For panel B, values are means ± SD of four replicate tank means per treatment (*n* = 4). Each tank mean was calculated by averaging the mean values of its four sampled fish.

Intestinal sections from NC showed epithelial rupture, whereas those from EAW groups had more intact mucosal folds and a more regular distribution of goblet cells, particularly in MD (Figure 1). Mucosal fold length did not differ significantly among groups. In comparisons of tank means, crypt depth was greater in LD and MD than in NC, and goblet-cell number was higher in HD than in NC (*p* < 0.05).

### 3.4. Metabolomic Alteration by Dietary EAW

**Figure 2:**
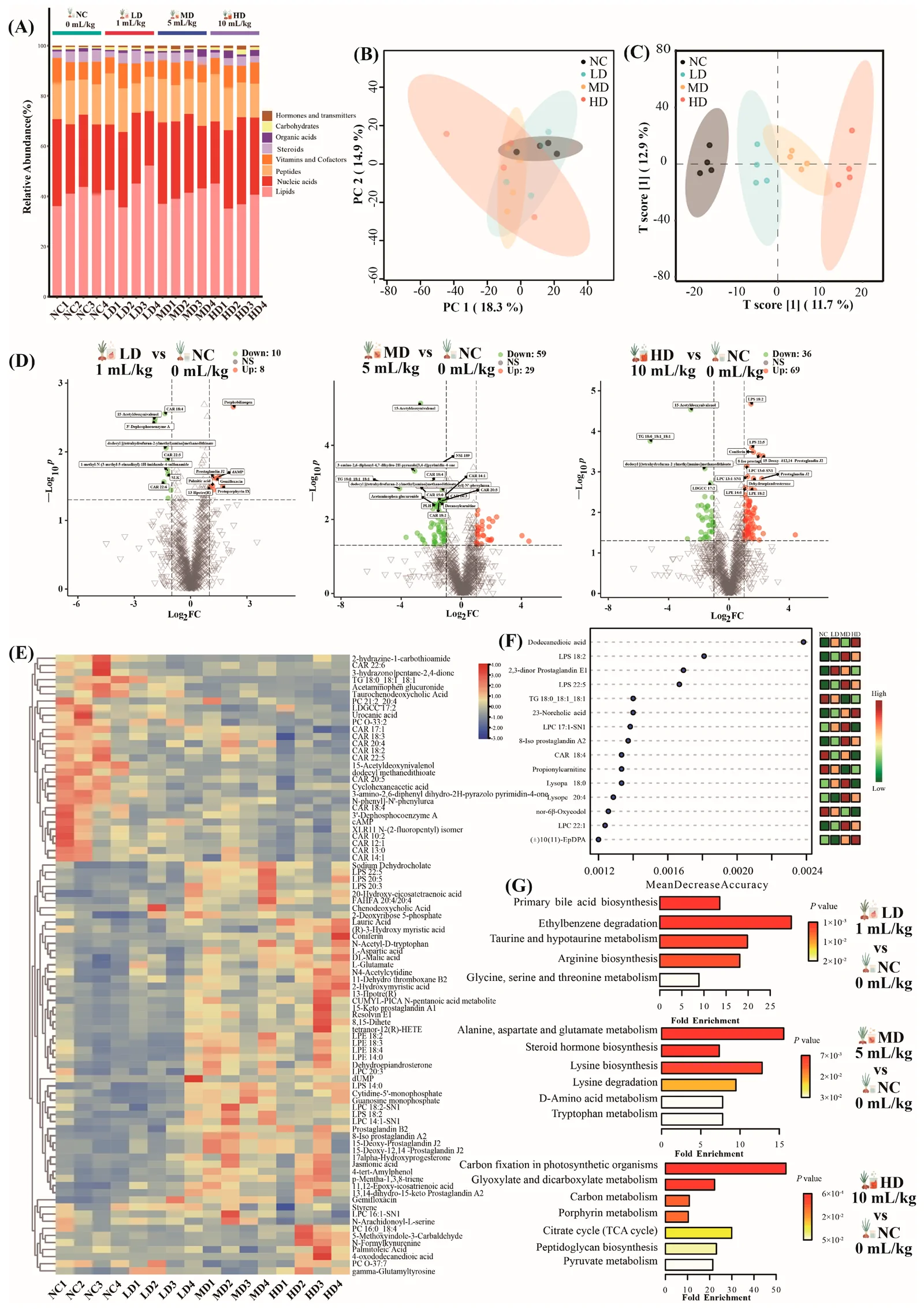
Metabolomic alteration by dietary EAW in the intestines of leopard coral grouper. (**A**): Classification of metabolites in the intestines of leopard coral grouper. (**B**): PCA score plot of metabolites. (**C**): OPLS-DA score plot of metabolites. (**D**): Volcano plots of intestinal DMs in LD vs. NC, MD vs. NC, and HD vs. NC groups. (**E**): Heatmap of DMs across all EAW groups. (**F**): Machine learning analysis of DMs using a random forest model. (**G**): KEGG pathway analysis of DMs in LD vs. NC, MD vs. NC, and HD vs. NC groups.

A total of 1363 metabolites were detected and identified. Lipid, nucleic acid, and peptide species were dominant components of the intestinal metabolome (Figure 2A). PCA indicated no clear separation among groups, whereas supervised OPLS-DA displayed separation between the EAW groups and NC (Figure 2B,C). Using *p* < 0.05 and |log_2_(Fold Change)| > 1, differential metabolite analysis identified 18 DMs in LD (8 upregulated and 10 downregulated), 88 in MD (29 upregulated and 59 downregulated), and 105 in HD (69 upregulated and 36 downregulated), each compared with NC (Figure 2D).

Several acylcarnitines were lower, whereas several lysophospholipids and prostaglandin-related metabolites were higher, in the EAW groups (Figure 2E). Random forest analysis ranked the top 15 metabolites by importance (Figure 2F). KEGG enrichment results included primary bile acid biosynthesis and taurine and hypotaurine metabolism in LD; alanine, aspartate and glutamate metabolism and steroid hormone biosynthesis in MD; and carbon metabolism and glyoxylate and dicarboxylate metabolism in HD, each compared with NC (Figure 2G).

### 3.5. Intestinal Microbiota Alteration by Dietary EAW

**Figure 3:**
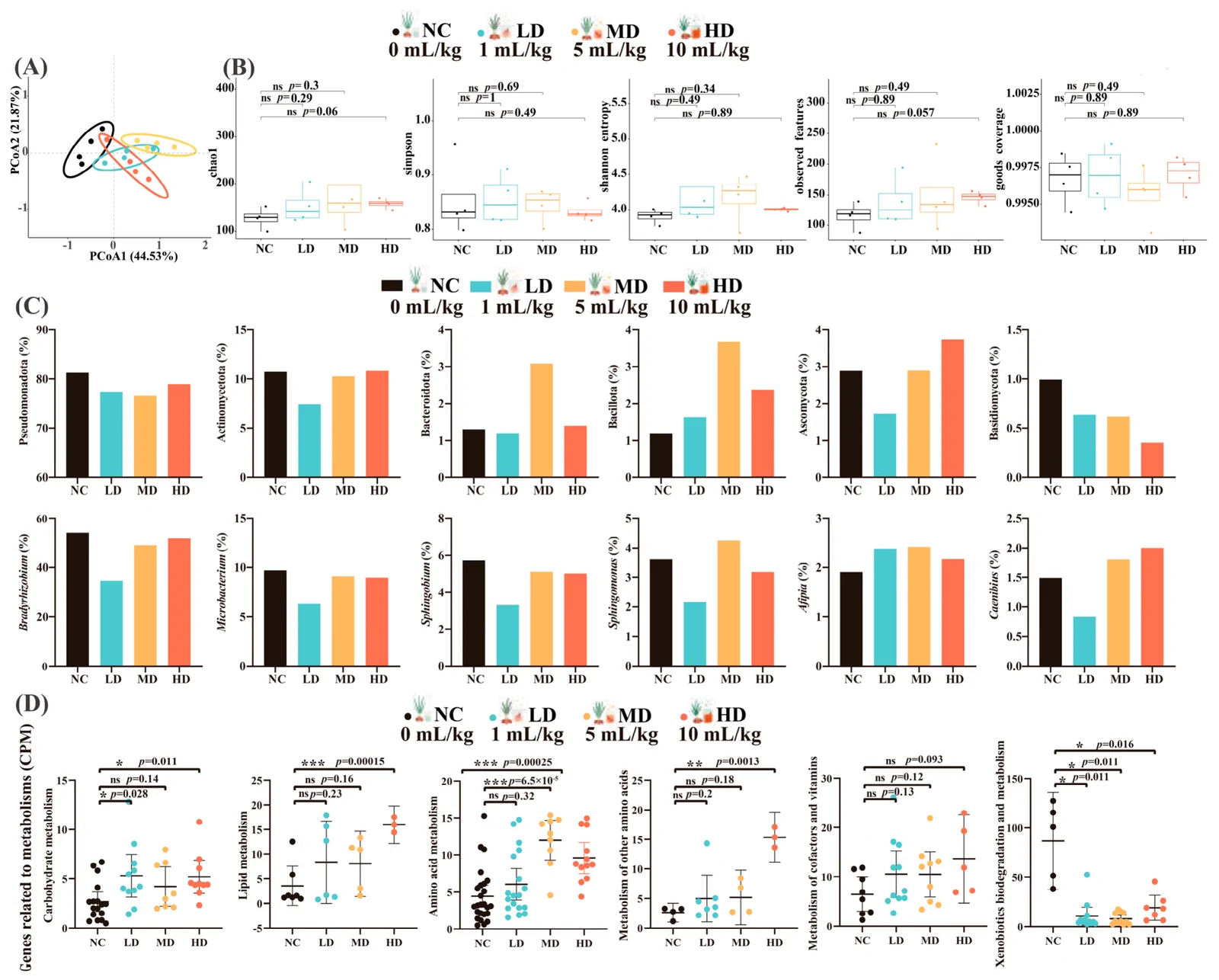
Intestinal microbiota composition and function alteration by dietary EAW. (**A**): PCoA of intestinal microbiota. (**B**): Alpha diversity analysis. (**C**): Relative abundance (%) of intestinal microbiota at the phylum and genus level. (**D**): Functional differences in metabolism-related pathways of intestinal microbiota in leopard coral grouper. ns, not significant (*p* ≥ 0.05); * *p* < 0.05; ** *p* < 0.01; *** *p* < 0.001.

PCoA visualized variation in microbial community composition (Figure 3A). Species richness and the Shannon, Simpson, and Chao1 indices did not differ significantly among treatments (*p* > 0.05; Figure 3B). The dominant phyla were Pseudomonadota, Actinomycetota, Bacteroidota, Bacillota, Ascomycota, and Basidiomycota. Relative abundances of Pseudomonadota and Basidiomycota were lower in EAW groups than in NC. Actinomycetota was lower in LD and MD, but not HD, whereas Bacteroidota and Bacillota were higher in MD. At the genus level, relative abundances of *Bradyrhizobium*, *Microbacterium*, and *Sphingobium* were lower and *Afipia* was higher in supplemented groups (Figure 3C).

KEGG functional profiling showed significantly higher abundance of carbohydrate metabolism-related genes in LD and HD than in NC. Amino acid metabolism genes showed higher abundance in MD and HD, while lipid metabolism and metabolism of other amino acids were higher in HD. Cofactor and vitamin metabolism genes did not differ significantly among groups (Figure 3D).

**Figure 4:**
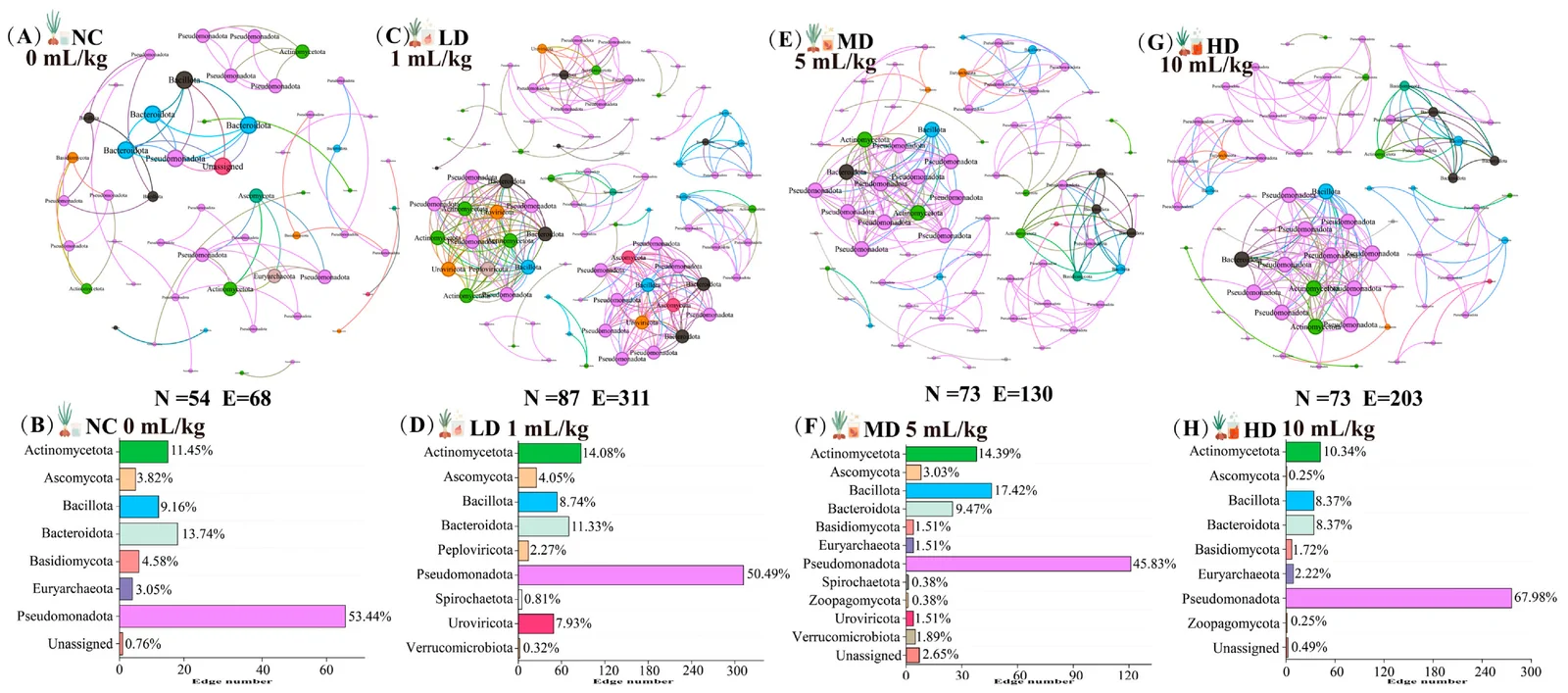
Co-occurrence network analysis of intestinal microbiota by dietary EAW. (**A**–**H**): Intestinal microbiota co-occurrence networks and phylum-level interaction edges of the NC, LD, MD, and HD groups, respectively.

Microbial co-occurrence networks showed more nodes and edges in the LD, MD, and HD groups than in NC (Figure 4). Pseudomonadota, Actinomycetota, Bacteroidota, Bacillota, and Ascomycota were dominant taxa in the networks.

### 3.6. The Interaction Between Intestinal Microbiota and Metabolites

**Figure 5:**
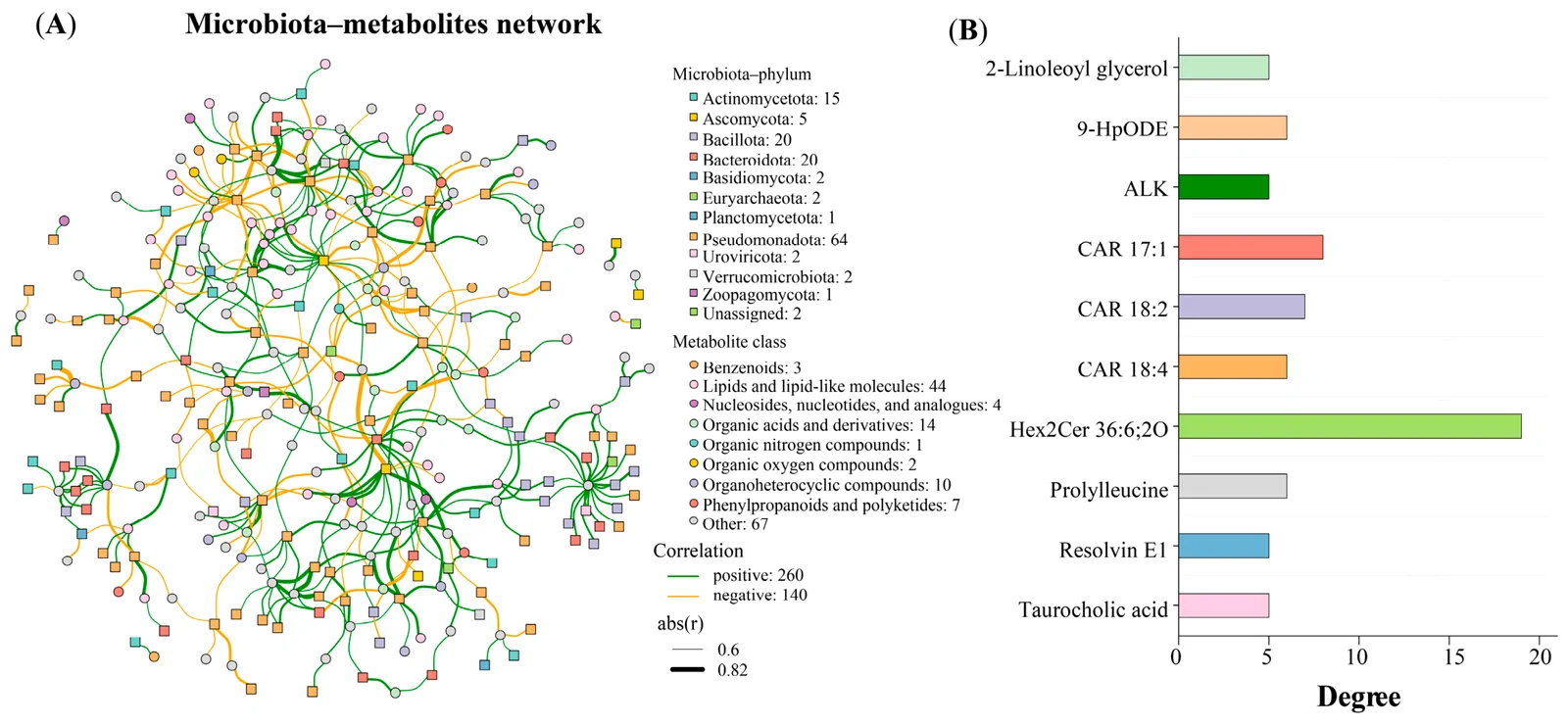
Correlation network of intestinal metabolites and microbiota in leopard coral grouper. (**A**) Significant correlations between metabolites and microbial taxa. Green lines indicate positive correlations, and yellow lines indicate negative correlations. Circles represent metabolites and squares represent microbial taxa. (**B**) Node degree of metabolites in the correlation network.

Spearman correlation networks linked intestinal microbial taxa, including Pseudomonadota and Actinomycetota, with lipid metabolites (Figure 5A). Hexosylceramides and carnitines were associated with multiple taxa and had high connectivity in the network (Figure 5B).

### 3.7. Multi-Regulatory Genes of EAW for Promoting Intestinal Health

**Figure 6:**
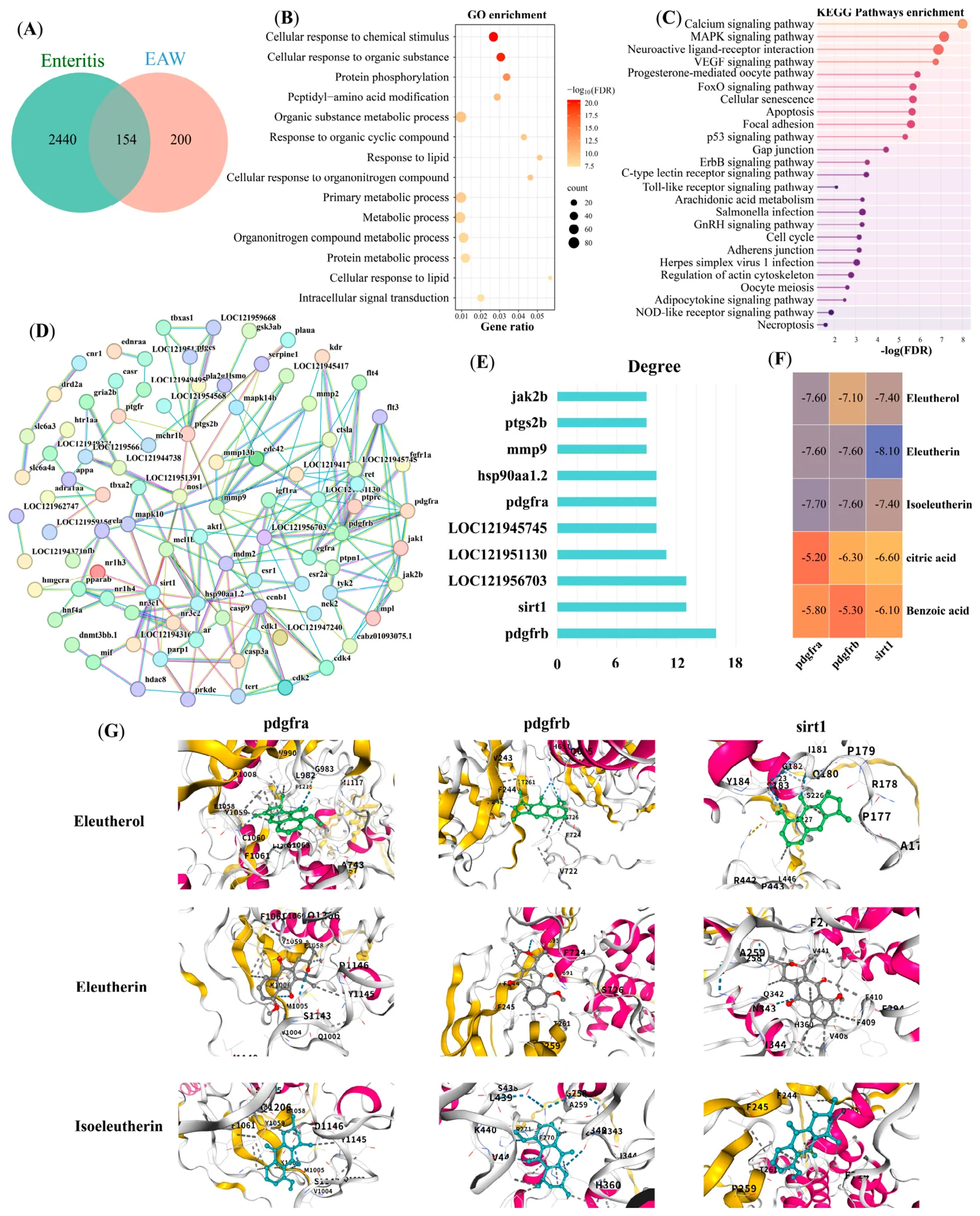
Network pharmacology and molecular docking of EAW in relation to enteritis. (**A**) Overlap between predicted EAW targets and enteritis-associated genes. (**B**) GO enrichment of the 154 shared target genes. (**C**) KEGG analysis of the 154 EAW–enteritis target genes. (**D**) PPI network of the 154 EAW–enteritis target proteins. (**E**) Node degrees of the top 10 targets in the PPI network; higher degree indicates connections with more targets. (**F**) Molecular docking scores (binding energy: kJ·mol^−1^). (**G**) Representative three-dimensional binding poses of eleutherol, eleutherin, and isoeleutherin in the binding pockets of Pdgfra, Pdgfrb, and Sirt1.

After duplicate targets were removed, 2594 enteritis-related targets were obtained from GeneCards, OMIM, and TTD. Of the 354 predicted EAW targets, 154 overlapped with the enteritis-related targets (Figure 6A). These shared targets were analyzed in STRING. GO enrichment included cellular response to organic substances, cellular response to lipids, and protein metabolic processes (Figure 6B). KEGG enrichment included calcium signaling, cellular senescence, and apoptosis (Figure 6C). The PPI network contained 154 EAW–enteritis targets (Figure 6D). Pdgfrb, Pdgfra, Sirt1, LOC121956703, and LOC121945745 were among the targets with the highest node degrees (Figure 6E).

Pdgfra, Pdgfrb, and Sirt1 were selected for docking with the main EAW components eleutherol, eleutherin, isoeleutherin, citric acid, and benzoic acid. The 15 successful component–target combinations all had negative docking energies (Figure 6F). The lowest-energy poses placed eleutherol, eleutherin, and isoeleutherin in the protein active pockets, with hydrophobic interactions as the primary binding mode (Figure 6G).

## 4. Discussion

EAW produced different responses in growth and survival in juvenile leopard coral grouper. Final body weight, weight gain rate, and specific growth rate were slightly higher in LD and MD than in NC, but these differences were not significant. Thus, this trial did not show a growth-promoting effect. Survival was significantly higher in MD and HD than in NC, with the highest value in MD. Histology, metabolomics, metagenomics, and network pharmacology were used to examine intestinal responses that might help explain this survival pattern.

The histological findings suggest that intestinal protection may have contributed to the survival differences. More intact mucosal folds and increased goblet-cell numbers are consistent with a mucosal barrier response. Goblet cells and mucus help maintain intestinal homeostasis [21].

Lower intestinal acylcarnitines suggest altered fatty-acid handling after EAW supplementation (Figure 2E,F). Acylcarnitine accumulation accompanies incomplete fatty-acid oxidation in mammalian muscle [22], and intestinal inflammation can impair epithelial use of these substrates [23]. Acetyl-L-carnitine also improved barrier function in murine colitis [24]. Together with the histological findings, these changes suggest that EAW may promote intestinal health by modulating carnitine metabolism and supporting epithelial energy balance and mucosal integrity.

Changes in lysophospholipids and prostaglandin-related metabolites may also contribute to the mucosal response. Intestinal phospholipid remodeling supports lipid absorption in mice [25], and dietary phospholipids improved digestive-enzyme activities and hindgut morphology in *P. leopardus* [26]. In mouse repair models, prostaglandin E2 (PGE2) promotes epithelial restitution [27], whereas microbial deoxycholate regulates later crypt regeneration by modulating PGE2 [28]. These studies link the observed lipid changes to processes involved in nutrient handling and mucosal renewal.

Pathway patterns varied with EAW inclusion, involving bile acid and taurine metabolism in LD, alanine, aspartate, and glutamate metabolism in MD, and central carbon metabolism in HD (Figure 2G). Lysine and lipid supply also influence growth and metabolic responses in *P. leopardus* [29,30]. These metabolic changes may help maintain nutrient utilization and mucosal renewal in the intestine.

Microbial community composition varied without a significant increase in alpha diversity (Figure 3A–C). Pseudomonadota and Basidiomycota were less abundant in supplemented groups, while Bacteroidota and Bacillota were more abundant in MD. Actinomycetota was lower in LD and MD, but not HD. Enteritis in *P. leopardus* is also associated with microbial, inflammatory, and metabolic disturbances [7], suggesting that these compositional changes may be relevant to intestinal health.

Responses differ across *Eleutherine* feeding studies. Selected doses improved growth in striped catfish [14], and bulb powder increased digestive-enzyme activities and microvillus density in white shrimp [15]. In Nile tilapia (*Oreochromis niloticus*) broodstock, 6.25–12.5 g/kg *E. bulbosa* powder produced greater growth than 25 g/kg, while Pseudomonadota increased [31], the opposite of its pattern here. These differences suggest that responses may depend on host species, life stage, preparation, and dose.

Carbohydrate- and amino-acid-related gene profiles suggest changes in microbial nutrient-use potential (Figure 3D). Associations of microbial features with carnitines and hexosylceramides link these profiles to lipid metabolism (Figure 5). Carnitine utilization can promote Enterobacteriaceae growth in cultures and murine colitis models [32], illustrating how this metabolite group may influence microbial composition. Larger co-occurrence networks contained more inferred taxon associations (Figure 4). Together with the mucosal findings, these results support intestinal protection as a possible contributor to the EAW-associated survival response.

Network pharmacology identified candidate host targets that may help explain these intestinal responses. *Eleutherine* constituents affect oxidative stress in infected mice and selectively modulate T-helper-cell responses [33,34]. Here, *pdgfra*, *pdgfrb*, and *sirt1* were hub targets in the predicted interaction network. PDGF-BB promoted proliferation and wound closure in cultured human colonic fibroblasts [35], linking PDGF signaling to intestinal tissue repair. SIRT1 activation reduced endoplasmic-reticulum-stress-associated epithelial apoptosis in experimental colitis [36], while intestinal epithelial SIRT1 deletion disturbed microbiota-dependent homeostasis in mice [37]. The predicted targets therefore have roles in tissue repair, cellular stress, and host–microbiota interactions that are relevant to intestinal protection.

Because intestinal samples were collected only from endpoint survivors, the metabolic and microbial profiles may reflect both responses to EAW and differences in survivor composition. Their temporal relationship to the survival response could not be determined. Studies of *E. americana* preparations and dietary *Eleutherine* supplementation in shrimp support their potential to modulate intestinal microbiota and promote host health [12,15,16]. Our intestinal findings are consistent with this potential. Pathogen challenges and direct immune-function measurements in future studies would help clarify the mechanisms of intestinal protection and determine whether the benefits extend to disease resistance.

## 5. Conclusions

**Figure 7:**
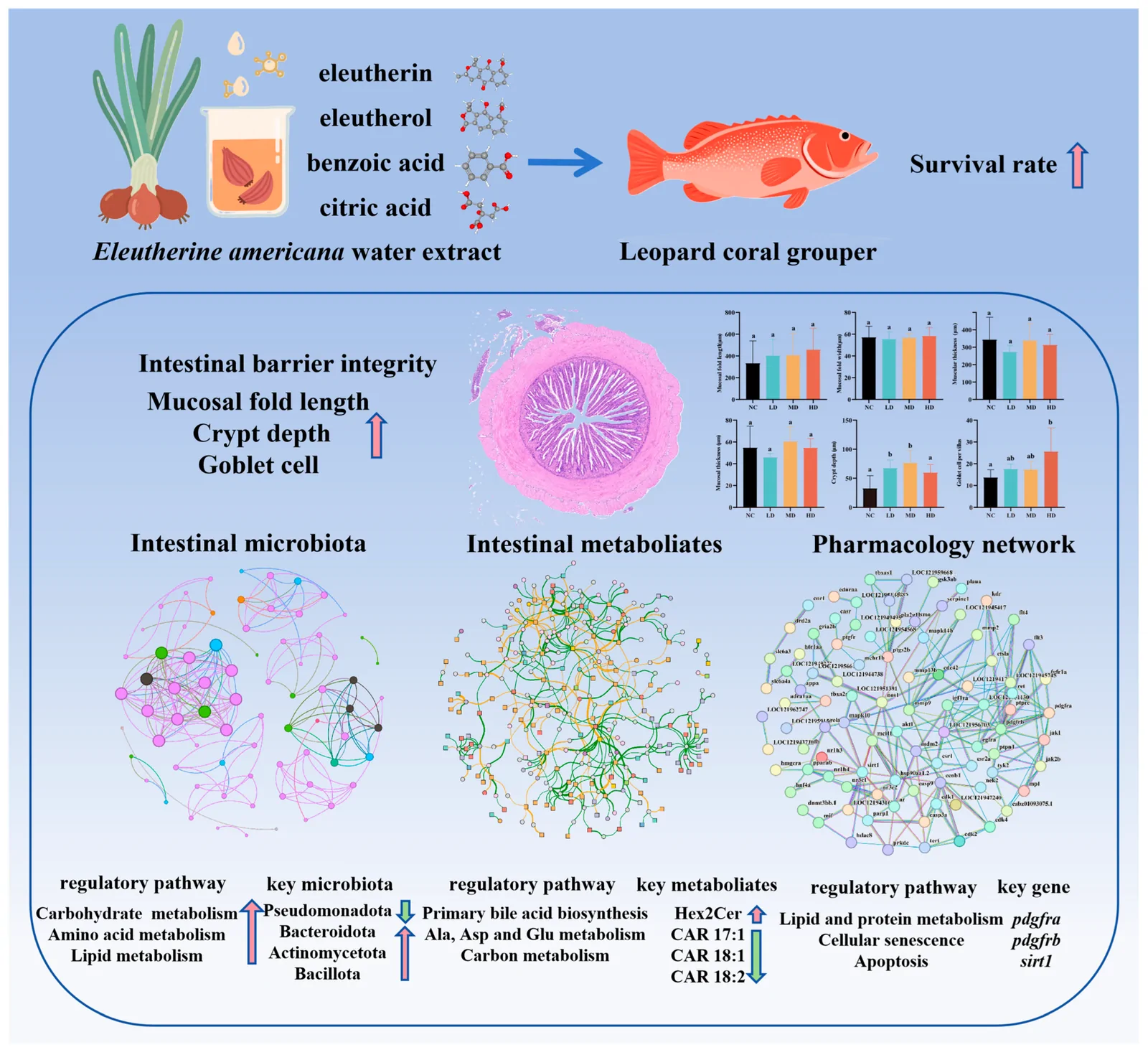
Schematic diagram of proposed mechanisms linking EAW supplementation to intestinal health through microbiota, metabolite, and pharmacology networks.

EAW supplementation was associated with higher survival and changes in intestinal morphology, metabolites, and microbiota among endpoint survivors. Network pharmacology identified *pdgfra*, *pdgfrb*, and *sirt1* as candidate targets through which EAW components may contribute to intestinal protection (Figure 7). Growth indices did not differ significantly, but the survival and intestinal findings support further evaluation of EAW as a functional feed additive for leopard coral grouper.

## Figures and Tables

**Table 1:** Effects of dietary EAW levels on growth performance of juvenile leopard coral grouper.

	Group			
	NC (0 mL/kg)	LD (1 mL/kg)	MD (5 mL/kg)	HD (10 mL/kg)
Initial mean body weight (g/fish)	11.72 ± 0.34 ^a^	11.72 ± 0.34 ^a^	11.72 ± 0.34 ^a^	11.72 ± 0.34 ^a^
Final mean body weight (g/fish)	38.46 ± 5.28 ^a^	39.86 ± 4.02 ^a^	43.53 ± 9.26 ^a^	37.87 ± 4.15 ^a^
Survival rate (%)	35.23 ± 11.95 ^a^	51.39 ± 5.32 ^ab^	69.44 ± 17.23 ^b^	59.72 ± 13.89 ^b^
Weight gain rate (%)	228.12 ± 45.01 ^a^	240.07 ± 34.31 ^a^	271.45 ± 78.49 ^a^	223.09 ± 35.44 ^a^
Specific growth rate (%)	1.97 ± 0.24 ^a^	2.03 ± 0.17 ^a^	2.15 ± 0.36 ^a^	1.95 ± 0.18 ^a^
Condition factor (g cm^−3^)	2.30 ± 0.17 ^a^	2.21 ± 0.18 ^a^	2.37 ± 0.22 ^a^	2.21 ± 0.23 ^a^

Note: Values are means ± SD (*n* = 4); initial and final weights are mean body weights per fish. Different superscript letters within a row denote significant differences (*p* < 0.05).

## Data Availability

Raw metagenomic sequencing data have been deposited in the NCBI Sequence Read Archive under BioProject accession number PRJNA1489476.

## Supplementary Materials

The following supporting information can be downloaded at: https://www.mdpi.com/article/10.3390/ani16213359/s1. Table S1: Nutrient levels of the basal diet (air-dry basis %). Table S2: The main chemical composition in EAW.
