# Supplementary material for: Modulatory Effects of Water Extract from Eleutherine americana on Intestinal Health: Insights from Growth, Morphology, Microbiota and Metabolomics in Plectropomus leopardus
Source: Animals (Basel). 2026 Oct 24;16(21):3359. doi: 10.3390/ani16213359 (PMC13761651; doi:10.3390/ani16213359)
Supplement: Supplementary file 1 [file animals-16-03359-s001.zip › animals-4614819-supplementary.pdf]

Table S1

Nutrient levels of the basal diet (air-fry basis %).

Nutrient levels	Content
Crude protein	49.20
Crude fatty	9.10
Crude fiber	1.90
Ash	15.6
Calucim	4.0
Total phosphorus	1.5

Table S2

The main chemical composition in EAW.

Number	Retention time	Ion model	Measured value (*m/z*)	Theoretical value (*m/z*)	Molecular formula	Name
1	1.12	$[M\text{-}H]^-$	191.0197	191.0197	$C_6H_8O_7$	Citric Acid
2	11.07	$[M\text{-}H]^-$	121.0296	121.0295	$C_7H_6O_2$	Benzoic acid
3	11.82	$[M\text{-}H]^+$	245.0805	245.0808	$C_{14}H_{12}O_4$	Eleutherol A/B
4	17.71	$[M\text{-}H]^+$	245.0810	245.0808	$C_{14}H_{12}O_4$	Eleutherol A/B
5	19.78	$[M\text{-}H]^+$	273.1123	273.1121	$C_{16}H_{16}O_4$	Isoeleutherin
6	20.11	$[M\text{-}H]^+$	273.1123	273.1121	$C_{16}H_{16}O_4$	eleutherin
7	21.72	$[M\text{-}H]^+$	245.0812	245.0808	$C_{14}H_{12}O_4$	Eleutherol C
